# Parametric Modeling of Biomimetic Cortical Bone Microstructure for Additive Manufacturing

**DOI:** 10.3390/ma12060913

**Published:** 2019-03-19

**Authors:** José A. Robles-Linares, Erick Ramírez-Cedillo, Hector R. Siller, Ciro A. Rodríguez, J. Israel Martínez-López

**Affiliations:** 1 Tecnologico de Monterrey, Escuela de Ingeniería y Ciencias, Ave. Eugenio Garza Sada 2501, Monterrey, N.L. 64700, Mexico; a01226825@itesm.mx (J.A.R.-L.); A00806274@itesm.mx (E.R.-C.); ciro.rodriguez@tec.mx (C.A.R.); 2 Laboratorio Nacional de Manufactura Aditiva y Digital (MADiT), Apodaca, N.L. 66629, Mexico; 3 Department of Engineering Technology, University of North Texas. 3940 N. Elm. St., Denton, TX 76207, USA; hector.siller@unt.edu

**Keywords:** 3D imaging, additive manufacturing, cortical bone, digital light processing, microstructure, parametric design, visual programming language, VPL

## Abstract

In this work we present a novel algorithm for generating in-silico biomimetic models of a cortical bone microstructure towards manufacturing biomimetic bone via additive manufacturing. The software provides a tool for physicians or biomedical engineers to develop models of cortical bone that include the inherent complexity of the microstructure. The correspondence of the produced virtual prototypes with natural bone tissue was assessed experimentally employing Digital Light Processing (DLP) of a thermoset polymer resin to recreate healthy and osteoporotic bone tissue microstructure. The proposed tool was successfully implemented to develop cortical bone structure based on osteon density, cement line thickness, and the Haversian and Volkmann channels to produce a user-designated bone porosity that matches within values reported from literature for these types of tissues. Characterization of the specimens using a Scanning Electron Microscopy with Focused Ion Beam (SEM/FIB) and Computer Tomography (CT) revealed that the manufacturability of intricated virtual prototype is possible for scaled-up versions of the tissue. Modeling based on the density, inclination and size range of the osteon and Haversian and Volkmann´s canals granted the development of a dynamic in-silico porosity (13.37–21.49%) that matches with models of healthy and osteoporotic bone. Correspondence of the designed porosity with the manufactured assessment (5.79–16.16%) shows that the introduced methodology is a step towards the development of more refined and lifelike porous structures such as cortical bone. Further research is required for validation of the proposed methodology model of the real bone tissue and as a patient-specific customization tool of synthetic bone.

## 1. Introduction

Biomimetics or the study of natural phenomena as a source of inspiration to solve problems has proved useful to understand and implement practical solutions. There are well-studied examples in materials sciences literature of the role that the organization of materials at such scale for nacre and bone has to determine the mechanical properties of the materials [1,2]. The principle of using a microstructure as a building block has shown to be a powerful strategy to achieve combinations of properties that are difficult to achieve with synthetic materials such as folding the strength and toughness, enabling structure-based color [3], enabling adhesive [4] or hydrophilic self-cleaning [5] surface capabilities. Even though historically autogenous implants of bone have been considered as the “gold standard” there is a gap for further development of multi-hierarchical nature inspired synthetic designs. The development of load-bearing orthopedic implants or void filling substitutes of bone has been foreseen as a promising process where design, manufacturing, cell culture, and implantation of cellular based structures (scaffolds) that can be implemented for bone tissue regeneration [6,7]. Cellular materials are generally characterized by large densification strains and high specific strength [8].

While prevalent implants for bone are based primarily on biocompatible solid implants made of stainless steel, cobalt-chrome or titanium based alloys, there are many potential advantages of employing non-metallic and porous structures such as the possibility to implement lighter and surface-enhanced materials [9]. Researchers have reported limitations on of metal based devices due to their low resistance to wear and fatigue post-implantation [10]. While rare hypersensitivity to metal has been attributed as a cause of aseptic loosening, pseudo-tumor formation and device failure are due to allergic reaction [11].

So far, most of the lattice structures have been made of metal alloys, but recent studies have been assessing polymers due to their versatility [12]. Moreover microfabrication using a layer-by-layer (additive manufacturing) approach has shown potential to add functionality with a low-cost approach [13,14,15]. However, the pursuit by the scientific community of alternatives to autologous or allogeneic bone grafts such as entirely synthetic bone graft strategies has focused on relatively simple lattices that do not recreate the convoluted and mutable essence of natural tissue. Shortcomings or abandonment of the development of lifelike models that are meticulous and versatile bone hampers the study of the conditions of the mechanical properties within the tissue through in-silico models and the ulterior manufacture of realistic bone tissue. Lifelike modeling could be employed for in-silico assessments of the fluid dynamics of the vascular system within the tissue, to examine the effects of stress and fractures over time and towards reducing the recovery time after an orthopedic intervention.

Cortical bone is a particular type of bone which sustains most of the mechanical loads in the body [16]. It possesses a complex structure, since its microstructure gives it anisotropic properties that vary from patient to patient, age and health [17]. As consequence, 3D modeling of bone at the micro-level is a challenging process.

Modeling tissues has been employed regularly at the macroscopic level. However advancements in microstructural imaging have revealed that properties of natural materials rely on their complex hierarchical structure [18]. Recent research in novel materials (e.g. composite structures) has been employed by imitating complex hierarchical organizations taking inspiration from nature. For instance, Tran et al. presented an algorithm to mimic the nacreous structure to form complex shapes that was tested numerically to compare the performance of the proposed structures against impulse loading [19]. A preliminary three-dimensional scaled model was implemented on the macroscale using additive manufacturing of ABS/PLA and ABS-tablet/Polyurethane-adhesive composites. Moreover, following the tendency of developing intricate and interactive design strategies design tools, Duro-Royo et al. [20] developed MetaMESH. The software is a computational design that combines scientific analytical methods and reverse engineering to generate a flexible protective surface based on an armored fish skeleton. The researchers scanned samples from deceased *P. seneagulus* specimen and developed a cell unit model based on parameters to populate structural regions of an exoskeleton.

Synthetic graft manufacturing has been boosted by additive manufacturing (AM) technologies which are inherently better suited to produce lattice-based structures that tackle with the clinical problems of autografts such as potential pain, morbidity and the difficulty to shape the implant to fit bone defects [21].

**Figure 1:**
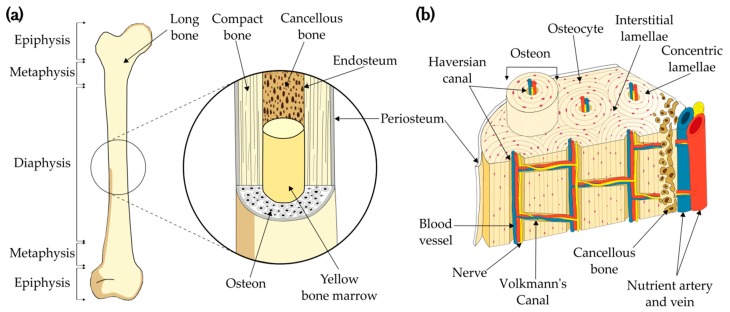
Schematic representation of an; (**a**) long bone and (**b**) microstructure.

Bone consists of two main tissues: the cortical layer in the outermost region and the cancellous or spongy layer filling the inner section of the bone. Cortical or compact bone can be regarded as a composite material made of unidirectional fibers called osteons immersed in an interstitial matrix [17] made of old surmineralized osteons [22]. Each osteon (100–250 μm in diameter) runs along the longitudinal axis of the bone and is surrounded by a cement line with a thickness between 1 μm and 5 μm [22] (see Figure 1a). The porosity of bone is attributed to two canal-based systems: Haversian canals (40–90 μm in diameter) run through the center of the osteons and the Volkmann’s canals (up to 50 μm in diameter), which are randomly oriented in a plane perpendicular to the Haversian canals [22] (see Figure 1b).

Both canal systems contain nerves, interstitial fluid, and the blood-transit arterioles, vessels and venules that all together permit the correct nutrition and performance of the bone [23]. Osteon density (i.e., osteon quantity per unit area) in human bones is within 10–25 osteons/mm^2^, and increment in osteon density has been shown to create an increment in the fracture toughness [24], which is proof that the mechanical integrity of long bone depends mainly on the osteon density. Nevertheless, the osteon density along with other parameters and properties (e.g., cement line thickness, elastic modulus, yield strength, porosity, osteon diameter, hardness) vary from person to person, depending on age and gender [25]. 

The present work aims to lay the foundations of biomimetic bone microstructure models that can be exploited with additive manufacturing. The study will begin by providing a brief outline of the current state-of-the-art of cortical bone modeling. Afterwards an in-depth analysis of the proposed parametric modeling based on a set of macrostructural and microstructural parameters is presented. The algorithm dynamically constructs a three-dimensional bone sample model composed based on a set of macro and microstructure parameters. The feasibility of manufacture has been evaluated using Digital Light Processing (DLP) to recreate healthy and osteoporotic cortical bone conditions as a proof-of-concept. The study is of relevance because the availability of an easy-to-use tool for the design of bone will bolster the possibility to implement polymer-based bone implants and provide means for detailed and tunable cortical bone tissue modeling. 

### 1.1. Bone structure

#### Cortical Bone as a Biomimetic 3D Model

Three-dimensional modeling of the cortical bone structure has been of interest in recent years for understanding the biomechanic behavior under different loading, cutting or fracture conditions to predict bone damage and minimize fracture risks.

Vergani et al. [26] developed a Finite Element Method (FEM) by two-dimensional modeling a quarter portion of a single osteon and studying the crack propagation from the interstitial matrix outside the osteon structure up to the center of the Haversian canal. However, the study was limited because isotropic properties were considered for the modeled microstructures and no consideration of Volkmann’s canals was included. Furthermore, crack propagation was only studied for transcortical loads and no consideration of axial forces was done.

Wang et al. [27] employed a two-dimensional model of cortical bone for fracture toughness and crack propagation simulations. The model was constructed applying statistical analysis to a set of microscopy images of a single female’s femur diaphysis. The results were limited to the single used bone, meaning that results are not applicable to another femur diaphysis. Additionally, the study was simplified to a bidimensional plane that did not include the influence of the Volkmann porosity.

Nguyen et al. [28] used a two-dimensional model of cortical bone to simulate the hydro-mechanical phenomenon that the tissue undergoes with a FEM considering different loading and fluidic (interstitial fluid) conditions. The experimental study was limited to a periodic arrangement of equally-sized osteons along a specified plane and concluded that the model employed should include three-dimensional features that considered the impact of Volkmann’s system.

Demirtas et al. [29] successfully modeled the cortical bone microstructure of different male donors in a three-dimensional space by getting 1.17 × 0.89 mm^2^ microscopical images of the bone samples, converting them into curves and ellipses using software and then extruding them out of plane by 1 mm. The study employed images acquired from a region of the bone from a microscope and projected the design in the remaining dimension to produce a homogenous structure.

Wang and Ural [30] developed a three-dimensional model of the mineralized collagen fibrils that compose the bone structure. They considered their alignment, arrangement and orientation along the bone to determine the impact that the fibrils have on the bone fracture mechanism. However, the control volume was of the range of the fibrils (60–100 nm diameter); hence, the impact of the overall arrangement of several osteons and the Volkmann’s and Haversian porosities were not studied and missed out on the three-dimensional modeling.

Khor et al. [31] modeled a full long bone’s cortical layer to predict fracture and bone response. The researchers used a Human Body Model (HBM) and a Finite Element Method (FEM) where properties were defined orthotopically in the radial and axial directions. Therefore, the model is only reliable for macro-level in-silico assessments (e.g., fracture, stress distribution). Additionally, their model cannot be used for fluid (i.e. interstitial fluid, blood) and cutting simulations because the model does not consider microchannels.

Predoi-Rancila et Crolet [32] developed SiNuPrOs (Simulations Numériques des Propriétés de l’Os) which is a mathematical model of the hydroxyapatite mass based on 28 parameters including physical, architectural and mineralization. The model was implemented in Matlab using basic cell discretization with a library of modular finite element library (Modulef). However, their model was limited in the variations of size and space of the different bone microstructures.

A recent work by Wu et al. [33] proposed and tested a novel topology optimization algorithm for manufacturing porous structure that mimics the porous infill of a femur bone (trabecular bone). The methodology employs local volume constraints to obtain optimized stiffness from porous structures. To verify the manufacturability the authors employed Fusion Deposition Modeling (FDM) to replicate models generated by the algorithm. 

Rapid prototyping (via AM) of scaffold for bone tissue replacement has recently been explored further by Gregor et al. [34]. Researchers focused on the design of scaffold structure for cell proliferation of osteosarcoma cells. The proposed modeling technique showed that Young´s modulus varied accordingly with the estimated porosity and that biofabrication was successfully developed for samples with porosity values between 30% and 50%. Bone is perfused by a sparse capillary network that allows for the exchange of nutrients/wastes between blood and the bone cells. The net movement of calcium ions to and from bone is critical for the proper functioning of nearly every organ. The Haversian system is the location where nutrient exchange occurs between the blood and the bone tissue [35]. 

Table 1 shows that customizable three-dimensional model that considers the Volkmann system and the Haversian systems has not been considered extensively, despite the importance of the microstructural characteristics on the overall mechanical properties. Insights derived from exploring the effect of fluid mechanics of biofluids remain limited by the availability of dynamic and adaptable models that can be incorporated into Computational Fluid Dynamics (CFD) assessments of the behavior of specimens during the manufacturing process and after the implantation. Multiscale and integral development of a biomimetic bone model has not been tackled to the extent required. Current attempts to digitally recreate the complex constituents of bone have either oversimplified or neglected elements of the bone. The majority of the efforts have focused on the recreation of particular specimens. Therefore, there is not a parametric modeling procedure reported that accurately represents a bone for a wide range of microstructural features. Most reported three-dimensional bone models are isolated for specific cases or purposes. 

## 2. Materials and Methods

### 2.1. Algorithm for Generating the Cortical Bone Models

The three-dimensional parametric design of cortical bone can be processed using a user-friendly free-form surface modeler CAD software (Rhino 6, McNeel, Seattle, WA, USA) and the built-in visual programming language (VPL) Grasshopper from the same provider. Coupling these tools can help build generative algorithms for models using standard design tools (point and curve sketching), transforming tools (rotate, mirror, bend) guided by a set of parameters settled from a script program. Notable reported examples of this approach can be found in a wide range of fields; i.e., in architecture with the parametric design of roof shells formed by repetitive modules of Catalan surfaces [36] or as a tool for the development of a parametric design platform of heart valve geometry for an isogeometric (IGA) analysis of a fluid-structure interaction [37].

**Figure 2:**
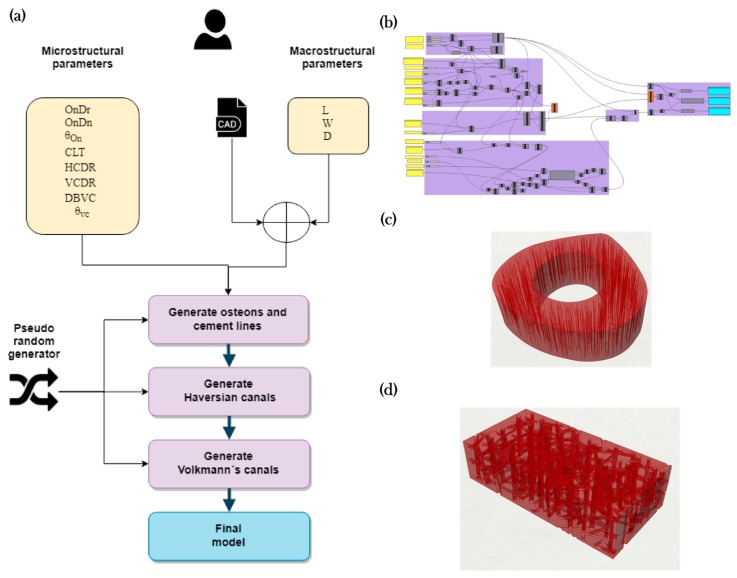
Bone modeling algorithm: (**a**) pseudocode; (**b**) screenshot of the VPL script; (**c**) final model bone shape, and (**d**) example of a model with rectangular prism.

Biomedical engineers, physicians or any end-user can select the macrostructural shape to be a rectangular prism or bone-shaped with any desired general dimensions. Figure 2a shows the general process of the algorithm and Figure 2b is a screen capture of the algorithm in the software. Figure 2c,d show two different bone structures that vary in shape and size.

A set of parameters are specified by the user including the length of the model, osteon density, osteon diameter range and inclination angles. To recreate the randomness within the different cellular domain in nature, randomness in the model is considered by adding pseudo-numbers (using seed values). After the part is created, the user can change the seed values of the pseudo-random numbers to generate different bone structures at the micro-level that have the same morphology characteristics between them in the macro-level. The user can export the model in several file formats that may be input into several software that allows an in-silico assessment to be performed.

#### 2.1.1. Modeling Algorithm

There are two kinds of parameters for the algorithm. The first corresponds to the geometrical (macrostructural) features of the bone (see Figure 2b). The user can either determine a rectangular prism dimensions or use a previously generated file with a free-form shaped bone (see Figure 2c). By default, the bone’s longitudinal axis is constructed along the Z vector of the software. If the user prefers to model a bone-shaped structure, the inner and outer curves of the cortical tissue must be the inputs and the box dimensions must be left at zero. If the user gives both box dimensions and curves, the algorithm will dismiss the curves and create a rectangular prism model according to the bounding box dimensions. Figure 2d shows an example of a model generated using these dimensions.

The second type of parameters corresponds to the nature of the microstructure of the bone (see Table 2). Each of these inputs is supplemented by one or two seeds for the pseudo-random number generation. These entities are used for the selection of a specific value of the input variable which is within the range specified.

#### 2.1.2. Modeling Algorithm Process and Steps

**Figure 3:**
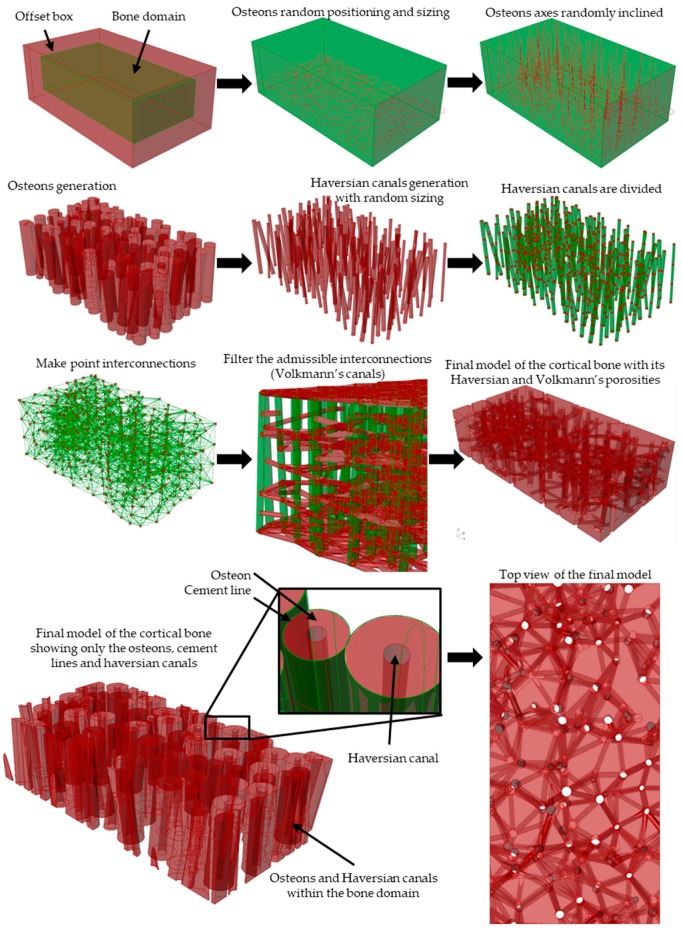
Step-by-step modeling process according to the algorithm.

A step-by-step explanation of the proposed algorithm is depicted in Figure 3. The program´s first task is to generate a solid three-dimensional volume. Afterwards, the lower base (or bottom) of the volume is filled with circles (representing the osteons) with a random size and position accordingly to the microstructural parameters (i.e., *OnDr*, *OnDn*, and seeds). Then, a vector is constructed from each circle center towards the top surface of the bone volume (this is considered with the osteon inclination angle range ($\theta_{On}$) and its respective seeds). A solid pipe-like cylinder is created along each vector with the same diameter as the base circle. The cement line is generated as a tangent concentric cylinder to the solid pipe and the thickness is determined with the seed values and the cement line thickness range (*CLT)*. The Haversian Canals are constructed along the same vectors using the diameter range (*HCDR*).

For the generation of Volkmann’s canals, the algorithm takes the distance between Volkmann’s canals (measured along the axis of the osteons) range (*DBVC*) value to set the position of equally-distanced points for each vector line. These interconnection points (IPs) are linked with other surrounding IPs with a straight line. Those lines that does not comply with the maximum inclination angle ($\theta_{VC}$) are omitted from the model. Then, using the seed values and the Volkmann’s canals diameter range (*VCDR*) each interconnection line is transformed into a solid pipelike cylinder that represents the Volkmann’s microchannels.

Finally, the bone model is constructed by merging and subtracting all the individually created structures using boolean and logic functions. The user can export the structures at different levels (e.g., only the osteons, only the cement lines, only the vascular porosities, whole bone model) and in multiple file formats.

#### 2.1.3. Modeling Algorithm In-Silico Validation with Porosity Check

To assess the generated three-dimensional models that mimic the cortical bone microstructure, a porosity check is included in the algorithm program. After the model is generated, the algorithm runs a volume check for each microstructure and provides the volume fraction of the Haversian canals and the Volkmann’s canals with respect to the total cortical bone volume. For a healthy person, the canals represent the 14 ± 6% of the total volume of the cortical bone, being 6 ± 3% from the Volkmann’s porosity and 8 ± 3% from the Haversian [22]. Therefore, if healthy-person morphologic and biologic characteristics are included in the algorithm inputs, the porosity check will assess that the final porosity ratios are congruent with those cataloged as healthy porosity values.

**Figure 4:**
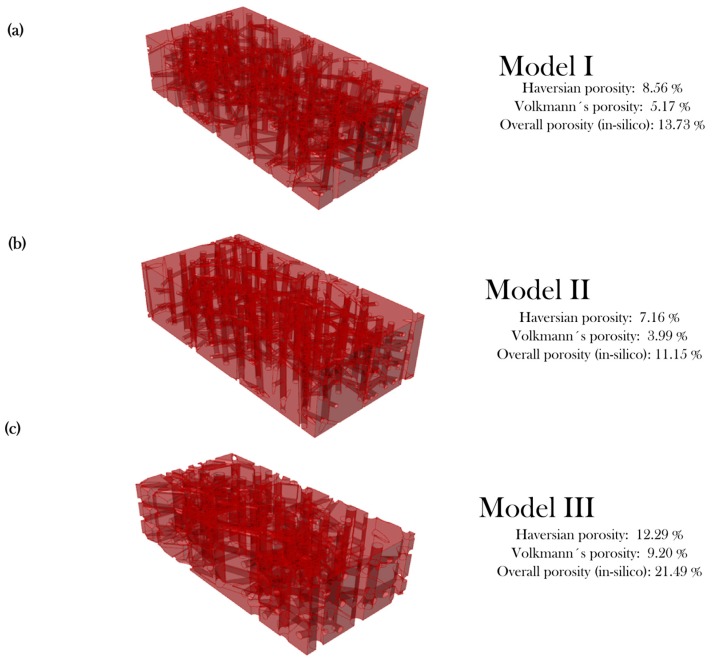
Models and porosity of the models used for algorithm validation. (**a**) Model I (healthy) exhibited a haversian porosity of 8.56%, Volkmann’s porosity of 5.17% and overall porosity of 13.73%, which is within limits of healthy bone structure. (**b**) Model II (healthy) exhibited a haversian porosity of 7.16%, Volkmann’s porosity of 3.99% and overall porosity of 11.15%, which is within limits of healthy bone structure. (**c**) Model III (osteoporotic) successfully exhibited out-of-range values in all porosities due to the out-of-range input values.

Three rectangular bone specimens (3 mm × 1.5 mm × 0.75 mm) were constructed with the algorithm using different sets of inputs (refer to Table 3 and Figure 4). The first two cases were considered for a healthy person and the third one for a person suffering osteoporosis. Model III input values are out of range concerning Table 1 for creating an osteoporotic structure that exceeds 20% of overall porosity.

### 2.2. Experimental Validation towards Scaffold Usage Employing Additive Manufacturing and XCT-Scanning

DLP was selected for manufacturing considering that the technology stands out, considering the better resolution (15 μm) to affordability ratio [38]. As an example, Two Photon Polymerization or TPP can operate within 100 nm resolution and allows for curing of shapes inside the resin bath and not just on its surface [39]. While the technology is commercially available (Nanoscribe [40]) the equipment can be still prohibitively expensive [41].

Initial assessment for manufacturing models directly from the CAD files was not possible due to the continuous clogging of the microchannels within the specimens.

Considering this technological limitation, scaled up versions (5×) of the Model I and Model III (see Table 3) in-silico- CAD models were used in an additive manufacturing process to create the specimens. Considering an isotropic scale of the dimensions, porosity remains unchanged.

A photopolymer resin (ABS-FLEX Black III) (product identifiers: hexane-1,6-diol diacrylate Isobornyl acrylate) (Envision TEC, Dearborn, MI, USA) which required a minimum energy of 700 mW/dm^2^ was used in a Digital Light Processing (DLP) apparatus (Envision Tec VIDA 371 HR, Envision Tec, Dearborn, MI, USA) with 1920 × 1080 pixels resolution and building volume of 96.88 × 50.62 mm.

A working resolution of 100 microns in the Z dimension was chosen for the specimens’ fabrication, considering the supplier’s recommendations for the selected material. While it is possible to work with layer thicknesses of 50 or even 25 microns, acrylonitrile butadiene styrene (ABS) was selected as the material with the best ability to absorb light and prevent deformation during curing. The equipment was loaded with the resin characterized by a post-cure tensile strength of 65 MPa and a Young’s Modulus of 1772 MPa according to the supplier.

The scaled-up versions of the Model I and Model III were manufactured with five replicas (12 specimens in total) under the same conditions (orientation of 90° respectively to the building platform) to avoid the clogging due to the small dimensions of the pores. Samples were printed with 100 μm of layer height and 5 mm of separation between the models in an array of 3 × 2. Since acrylate monomers have high photo initiator concentrations, DLP was used for fast polymerization and then post cured in oven is used for complete polymerization [42]. Specimens were processed with alcohol and half of the samples were exposed to UV light for 2 cycles of 1 min each one in a PCA 100 a light photo-polymerization chamber with a light delivered in a 360–420 nm wavelength range (Envision TEC, Dearborn, MI, USA) to reduce the anisotropic effect of the AM process [43].

Each sample was scanned using an XCT-micro scanner (Bruker Skyscan micro-ct 1172, Bruker, Kontich, Belgium), using x-rays with a setup of 55 kV voltage, a current of 18mA, 10 watts of power, and 120 μs of exposure time. The micro-ct images were obtained without filter and with a full rotation of 360° of the samples. Files were processed with CT analyzer 1.18.4.0 (Bruker, Kontich, Belgium), and rendered with Avizo 9.20 (Thermo Fisher Scientific, NH, USA). The porosity check was performed by measuring the solid and empty areas in each layer of the scan. Intended porosity (biomimetic bone designed with the algorithm) and dimensional precision were also evaluated with a scanning electron microscope (SEM) EVOMA25 (Carl Zeiss, Jena, Germany) with an acceleration voltage of 10 kV.

Specimens generated with additive manufacturing (DLP) were exposed to the X-rays to reconstruct a three-dimensional model using Solidworks (Dassault Systèmes, Vélizy-Villacoublay, France) as a 3D image surface rendering tool. A region of interest (ROI) and the volume of interest (VOI) was defined to select a fraction of the volume to be mesh and formatted into a native stereolithography CAD file (STL). The ratio of the solid mass volume and VOI was obtained to define a resultant specimen porosity.

## 3. Results and discussion

### 3.1. In-Silico Porosity Check

Figure 4 shows the different microstructures that were created with the algorithm with their respective porosity check values. Model I (healthy) showed a Haversian porosity of 8.56% and a Volkmann porosity of 5.17% (13.73% overall porosity). Model II (healthy) exhibited 7.16% and 3.99% for the Haversian and Volkmann porosities, respectively, which adds to a total of 11.15%. Both models are within the acceptable limits of porosity stated in literature. Model III (osteoporotic) consisted of a 12.29% Haversian porosity and a 9.20% Volkmann porosity (both values out of range), exceeding the total healthy porosity of 20% by having an added value of 21.49%. The modeled microstructures were successfully developed within limits according to the permissible ranges of porosity.

The overall porosity for the three in-silico models shows that employment of the VPL paired with reported values of the microstructure can produce geometrical models without employment of images acquired from human tissue. While the evaluation of the degree of precision of the model with a real tissue is hampered by the intrinsic nature of cortical bone anatomy and morphology, the porosity check is a fair indicator that shows a good fidelity of biomimicking.

### 3.2. Experimental Porosity Check

To assess the feasibility of the proposed methodology towards manufacturing polymer-based bone substitute materials, we employed the CAD files produced by the algorithm with the more discernable features (Model I and Model III) to manufacture and characterize specimens produced with additive manufacturing. This provides a tangible proof-of-concept physical component with the designated bone porosity. DLP was selected as the most viable technology considering. 

**Figure 5:**
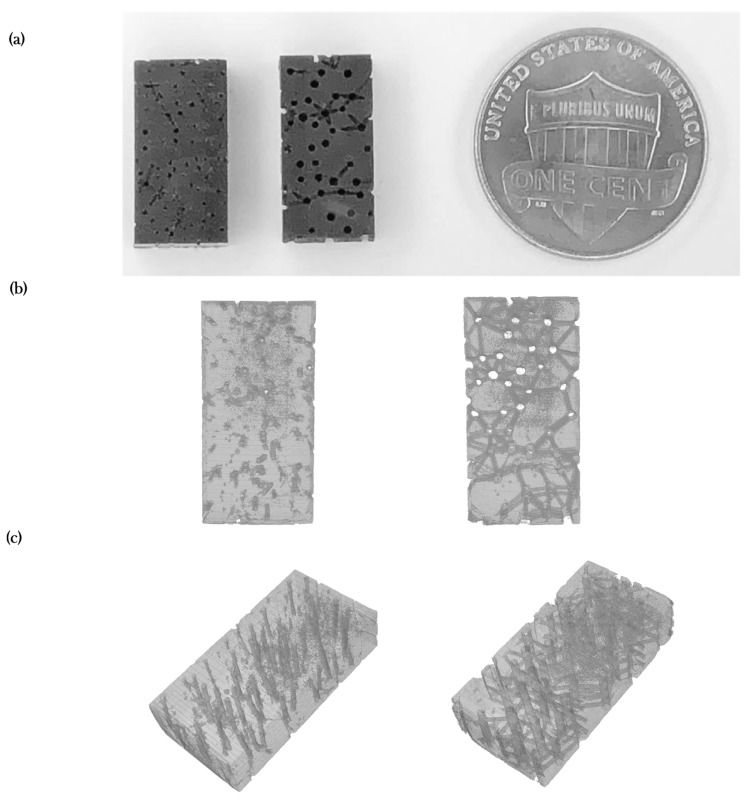
Additive manufacturing assessment; (**a**) Photos of the specimens manufactured using DLP for the scaled up versions of Model I (**left**) and Model III (**right**); (**b**) MicroCT reconstructions (top view) of the scaled up versions of Model I (**left**) and Model III (**right**); (**c**) MicroCT reconstructions (isometric view) of the scaled up versions of Model I (**left**) and Model III (**right**).

Figure 5a shows the fabricated specimens with the specified AM technology. Figure 5b,c show images of the three-dimensional models produced from the X-ray Computer Tomography (XCT) system. The images show that the reconstruction of the Haversian and Volkmann canals is more defined in the osteoporotic structure, meaning that the definition of micro-tunnels and interconnections can be observed and measured more in the osteoporotic model (Figure 5b,c).

Table 4 shows the average porosity for the in-silico and experimental models. A difference on the overall porosity up to 7.94% was found. 

**Figure 6:**
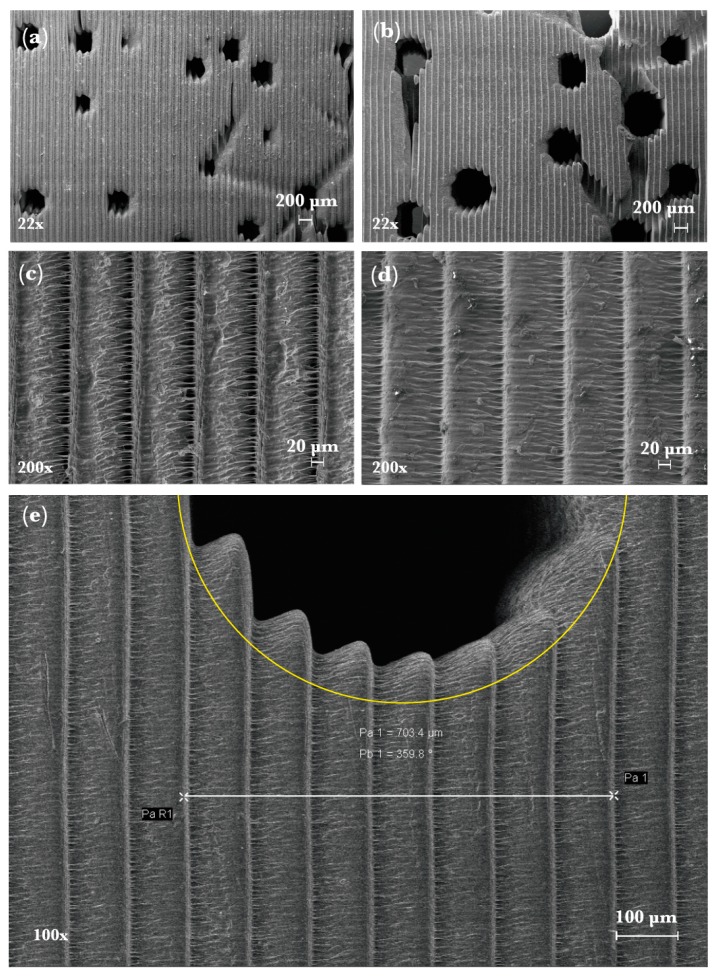
SEM micrographs of top lateral surfaces of Model I and Model III. (**a**) Model I, (**b**) Model III; (**c**) image of layers of a model before curing; (**d**) image of layers after curing; (**e**) designed pore in yellow and the printing result for Model III.

Figure 6a,b show SEM micrographs of the healthy and osteoporotic models. Figure 6c,d shows layers before and after curing. Differences between the intended porosity and the experimental porosity could be attributed to the restrictions of the resolution (100 µm) and inherent limitations of DLP.

The degree of the porosity of the models was analyzed before and after curing and estimated below <1% due to better addition, shrinkage or thermal expansion of the layers [43]. During characterization it was confirmed that pores and microchannels were found to be consistently larger (4.8%) for the osteoporosis model (Model III). Figure 6e shows an example of deviations between the designed porosity and the manufactured result where differences in one direction are composed by 7 layers (measured 703.4 μm). The difference between the virtual models and the manufactured specimens can be attributed to the reduced size of the microchannel and the limited resolution of the AM process (100 μm) for this material. While further research is required regarding the sources and reduction of the deviational error on manufacturing on biomimetic bone mode, the ability to reproduce the intended condition on the specimen (healthy or osteoporotic) was evaluated to be between 4% considering a threshold deviation as the difference of the measured average porosity to the nearest reported admissible value for the tissue condition (see Table 4).

## 4. Conclusions

The concluding remarks of this work are summarized as follows:

- A flexible parametric algorithm for mimicking the bone microstructure in a 3D model was successfully developed and employed for the first time. This approach allows the dynamic generation of a tissue model without exposing a patient to x-ray and can be adapted to different health conditions.
- Use of bone parameters and pseudo-random numbers allows the generation of bone microstructures dynamically. It was found that the algorithm is consistent for creating bone samples within acceptable porosity levels if the provided inputs are within healthy parameters. Conversely, including parameters outside those reported for healthy tissue will generate osteoporotic bone microstructure.
- Merging of two or more osteons is a feature of the algorithm. This represents a more realistic approach of intricate hierarchical structure of bone as osteon merging is naturally occurs during the bone modeling and remodeling process.
- 3D printing microstructural porous structures towards cortical bone implant fabrication remains a challenge given the limitations of additive manufacturing of microchannels for synthetic bone graft based on polymer. Implementation of technologies as TPP could increase the chance to reproduce biomimetic models with a 1:1 scale and remains a next step for future work.
- This work is one step forward in the modeling and fabrication of cortical bone.

## Figures and Tables

**Table 1:** Recent advancements in modeling and fabrication of bone microstructure.

Work	* Ref	Integrity	Volkmann System	Haversian System	Source	Dimension
Vergani et al.	[26]	Partial	NA **	Yes	Literature	2D
Wang et al.	[27]	Full	NA	Yes	Specimen	2D
Nguyen et al.	[28]	Partial	NA	Yes	Literature	2D
Demirtas et al.	[29]	Full	NA	Yes	Specimen	3D
Wang et al.	[30]	Partial	NA	NA	Literature	3D
Khor et al.	[31]	Full	NA	NA	Specimen	3D
Predoi-Racila et Crolet	[32]	Full	Yes	Yes	Literature	3D
Wu et al.	[33]	NA	NA	NA	Mathematical	3D
Gregor et al.	[34]	Full	NA	NA	Mathematical	3D

* Reference, ** Not applied.

**Table 2:** Reported microstructural parameter values for healthy bone tissue.

Parameter	Type	Description	Admissible Values
OnDr	Input	Osteon diameter range	100 to 250 μm [3,4]
OnDn	Input	Osteon density	10 to 25 Osteons/mm^2^ [24]
$\theta_{On}$	Input	Osteon inclination angle range	0° to 15° [23]
CLT	Input	Cement line thickness range	0 to 5 μm [22]
HCDR	Input	Haversian canals diameter range	40 to 90 μm [22]
VCDR	Input	Volkmann’s canals diameter range	40 to 50 μm [22]
DBVC	Input	Distance between Volkmann’s canals	150 to 500 μm [22]
$\theta_{VC}$	Input	Maximum inclination angle of the Volkmann’s canals	15° [23]
–	Output	Haversian porosity	6 ± 3% [22]
–	Output	Volkmann´s porosity	8 ± 3% [22]
–	Output	Overall porosity	14 ±6% [22]

**Table 3:** List of inputs for the models used for the algorithm in-silico validation.

Input Variable	Model I – Healthy	Model II – Healthy	Model III – Osteoporotic
OnDR (μm)	100–250	120–240	180–250
OnDn (Ons/mm^2^)	22	18.5	9.5
$\theta_{On}$ (°)	0–10	0–6.5	0–3
CLT (μm)	0–5	1–4	0–3
HCDR (μm)	50–90	60–85	95–150
VCDR (μm)	40–50	45–50	70–80
DBVC (μm)	150–500	150–400	165–400
$\theta_{VC}$ (°)	15	13	15

**Table 4:** Overall porosity for in-silico and experimental cortical bone models.

Model	Literature	In-silico	Experimental (Cured)	Threshold Deviation ^1^
I—Healthy	14 ± 6% [22]	13.73%	5.79 ± 0.64%	−2.21%
III—Osteoporosis	>20% [22]	21.49%	16.16 ± 1.02%	−3.84%

^1^ Calculated as the difference of the average porosity to the nearest reported value for the tissue condition.
